# Understanding informal payments in health care: motivation of health workers in Tanzania

**DOI:** 10.1186/1478-4491-7-53

**Published:** 2009-06-30

**Authors:** Silvia Stringhini, Steve Thomas, Posy Bidwell, Tina Mtui, Aziza Mwisongo

**Affiliations:** 1 Centre for Global Health, Trinity College Dublin, Dublin, Ireland; 2 INSERM U687, Hôpital Paul Brousse, Villejuif, France; 3 National Institute for Medical Research (NIMR), Dar Es Salaam, Tanzania

## Abstract

**Background:**

There is growing evidence that informal payments for health care are fairly common in many low- and middle-income countries. Informal payments are reported to have a negative consequence on equity and quality of care; it has been suggested, however, that they may contribute to health worker motivation and retention. Given the significance of motivation and retention issues in human resources for health, a better understanding of the relationships between the two phenomena is needed. This study attempts to assess whether and in what ways informal payments occur in Kibaha, Tanzania. Moreover, it aims to assess how informal earnings might help boost health worker motivation and retention.

**Methods:**

Nine focus groups were conducted in three health facilities of different levels in the health system. In total, 64 health workers participated in the focus group discussions (81% female, 19% male) and where possible, focus groups were divided by cadre. All data were processed and analysed by means of the NVivo software package.

**Results:**

The use of informal payments in the study area was confirmed by this study. Furthermore, a negative relationship between informal payments and job satisfaction and better motivation is suggested. Participants mentioned that they felt enslaved by patients as a result of being bribed and this resulted in loss of self-esteem. Furthermore, fear of detection was a main demotivating factor. These factors seem to counterbalance the positive effect of financial incentives. Moreover, informal payments were not found to be related to retention of health workers in the public health system. Other factors such as job security seemed to be more relevant for retention.

**Conclusion:**

This study suggests that the practice of informal payments contributes to the general demotivation of health workers and negatively affects access to health care services and quality of the health system. Policy action is needed that not only provides better financial incentives for individuals but also tackles an environment in which corruption is endemic.

## Background

There is growing evidence that informal payments are, in many low- and middle-income countries, the main source of health care financing [1]. In this paper, informal payments will be considered as unreported or unregistered illegal payments that have been received, in cash or in kind, in exchange for the provision of a service (or of a faster or better service) that is officially free [2-4].

In Poland, informal payments have been estimated to contribute to "as much as double of the physician's salary" [5]. In Bangladesh, earnings from unofficial charges exceeded the official salary by a factor of 10 [6], and by a factor of five in Cambodia [7]. Recent studies have estimated that unofficial fees constitute 10% to 45% of total out-of-pocket expenditures for health care in the countries considered [2,7-9]. Evidence of the existence of informal payments has been found in at least 22 studies [3], almost all referring to low-income countries.

Even if informal payments take the same form as official fees, as pointed out by Gaal [3] they could be "the worst possible form of private financing". First, they create a barrier to the accessibility of health services [9-15], which could affect the poor even more than official fees, due to the unavailability of exemption policies [3] and the arbitrariness of the payment [16]. Second, informal payments compromise the efficiency of health care provision by directing resources to services that are more profitable, rather than to those most effective [17], and to patients who are more profitable, rather than to those most in need [18]. Third, they create perverse incentives that potentially represent an obstacle to health policy [10,16,17]. Finally, the informal nature of unofficial payments undermines governments' ability to raise finances for health [1,2] and to regulate the financing of health care.

On the other hand, some authors have suggested that part of "these payments can be regarded as cost-contributing since they may ensure that staff receive their reservation wage and stay in employment" [1,19]. In addition, sometimes patients may pay for improved services [1].

Health worker motivation and retention are increasingly being considered a crucial response to the human resources for health crisis, especially in low- and middle-income countries [20,21]. In fact, the decision of health workers to migrate to other countries or take employment in the private sector is not due solely to demotivation caused by insufficient salaries and extreme working conditions, but is also influenced by the difficult environment remaining workers face, having to manage staff shortages resulting from health worker migration [22,23]. A number of studies have confirmed the predominance of financial incentives in determining health worker motivation and performance [24]. In fact, the relationship between inadequate salaries and the seeking of opportunities to raise earnings through charging informal fees has been strongly suggested [1,13,25,26].

The relationship between motivation, retention and seeking additional income in the public sector in the form of informal charges may not be straightforward. The use of such a practice can cause rivalries among health workers due to the competition for receiving payments, as well as the feeling of guilt and general discomfort [11]. Informal payments can demotivate health workers more than motivate them, especially in rural areas where patients are poor and for lower cadres of workers who do not receive a high share of the payments [11]. The extent to which each of these two possible relationships, positive or negative, apply has not yet been sufficiently investigated.

A clear understanding of the impact of informal payments on the health system has not yet been reached. It is clear that they represent a failure of the system, but Gaal's (2006) argument that an available service still guarantees better access to health care than no service at all needs reviewing. For this reason and given the context of a global health workers crisis [20], the specific relationship between informal payments, motivation and retention of health workers needs to be urgently investigated. Consequently, the authors explore the linkages between motivation, retention and informal payments by means of a case study from Tanzania.

### Background on Tanzania

The human resource crisis is particularly severe in Tanzania, as a consequence of restrictive government policies in the last few decades that have resulted in a freeze in recruitment of health workers [27]. The country suffers from a critical staff shortage, particularly in rural areas. Table 1 reports some key indicators of health and health services in Tanzania. In 2002, there were four nurses/midwives per 10 000 population, and there were only 822 physicians for the whole country [28,29].

**Table 1:** Selected health and health system indicators of Tanzania in 2002

Births attended by skilled health personnel (%)	46
Number of physicians	822

Density of physicians per 1000 population	1.7

Number of nurses and midwives	13 292

Density of nurses and midwives per 1000 population	27.4

Number of other health workers	29 722

Density of other health workers per 1000 population	61

Total expenditure on health as % of gross domestic product	4.7

Per capita government expenditure on health at average exchange rate (USD)	7

Out-of-pocket expenditure as % of private expenditure on health	81

Source: WHO, Country health system fact sheet 2006.

Apart from the traditional categories of health workers (doctors, nurses, midwives), in the Tanzanian health system there are several non-traditional cadres that do not hold internationally recognized qualifications. Among these are Clinical Officers and Assistant Medical Officers, who serve as doctors, and Nurse Aides and Medical Attendants as substitutes for nurses. These non-traditional cadres are also paid lower salaries than their traditional counterparts.

Health worker motivation is a central factor in retaining existing health workers and increasing the attractiveness of the professions [30]. In addition, the practice of charging informal payments in the health care system seems to be fairly common in the country [11], but its relationship with motivation and retention has not been fully explored.

The study had three objectives: first, to assess whether, how and in which ways informal fees occur in the case study area of Tanzania and represent a critical issue for the health system in the opinion of health workers; second, to assess whether and how informal earnings help boost health worker motivation; third, to understand in what ways informal payments might contribute to health worker retention in public health facilities.

## Methods

The phenomenon of informal payments is extremely complex and sensitive. The approach taken in the study has been qualitative to allow for this. As pointed out by Miles and Huberman [31], with qualitative research: "the researcher attempts to capture data on the perceptions of local actors from the inside [...] and suspend [...] preconceptions about the topics under discussion". In addition, qualitative research enables the researcher to "explicate the ways people in particular settings come to understand, account for, take action, and otherwise manage their day-to-day situations" [31].

Since the practice investigated is illegal, the use of focus groups was preferred, for their ability to explore a difficult and sensitive topic [32]. In fact, focus group discussions allow the participant to withhold self-incriminating information, something that might not have been possible with the use of in-depth interviews.

The research was carried out in the district of Kibaha, Pwani region. This district is relatively close to the commercial capital of Dar es Salaam but is still mainly rural. Health facilities at different levels in the health system were selected. The district hosts only one regional hospital (Tumbi) and one district health centre (Kibaha Health Centre), both of which have been included in this study.

The study was originally expected to include at least two health dispensaries; however, due to the small number of health workers in the dispensaries, it was possible to hold the FGD only by stopping all other activities. This would have been unethical, and so the study includes only one dispensary. Health workers to be included in the study were selected randomly from a staff list that was stratified by cadre.

All focus groups discussions were recorded with the permission of participants, according to best practice [31], and field notes were taken. Discussions were transcribed in KiSwahili and then translated into English.

All data were processed and analysed by means of the NVivo software package. Discussions were coded following the technique described by Strauss and Corbin [33], adapted to the use of Nvivo. A list of the codes used is provided in Additional file 1. Data were reviewed line by line and categories (Tree Nodes) were attributed to key concepts. During the process, subcategories were identified and subcodes (Child Nodes) were added. All the codes were created while keeping in mind the research questions. Two out of nine documents were checked to ensure that code-recode consistency was above 90% [31]. After coding all documents, each Tree Node was reviewed separately to check the appropriateness of the content.

## Results

In total, nine focus groups were conducted and where possible groups were divided by cadres. Due to this requirement, six out of nine focus groups were held in Tumbi regional hospital, which had a large number of employees available for each cadre, allowing the research team to conduct the focus groups without stopping routine activities. Furthermore, when more than one group was to be held within the same cadre, at least one was composed of females only.

Clinic managers acted as gatekeepers, inviting the participants to the focus groups. Seventy health workers were approached, of whom 64 (91%) participated in the focus group discussions (81% female, 19% male). Focus group size varied between six and 10 participants. In total 22 midwives, 14 nurses, 11 clinical officers, 10 medical attendants and 7 doctors took part in the study. Table 2 illustrates the composition of the focus groups.

**Table 2:** Composition of focus groups

	**Participants**	**Cadre**	**Gender**	**Location**
Group 1	6	Nurses	1M, 5F	Tumbi

Group 2	6	Midwives	6F	Tumbi

Group 3	6	Nurses	6F	Tumbi

Group 4	6	Midwives	6F	Tumbi

Group 5	6	Medical Doctors	5M, 1F	Tumbi

Group 6	7	Clinical Officers	5M, 2F	Tumbi

Group 7	10	Mixed	10F	Dispensary

Group 8	10	Midwives	10F	Health Centre

Group 9	7	Med. Attendants	6F, 1M	Health Centre

Focus group discussions lasted between one and two hours, depending on the size of the group and of the cadre. In general, it was observed that the lower the cadre, the longer the duration of the discussion. Groups composed only of females tended to develop better group dynamics than mixed ones, confirming the literature on the topic [34]. All focus groups apart from one were carried out in Swahili, with the help of a local researcher. In the focus group held in English, it was observed that participants were much less likely to participate and become involved in group discussions. For this reason, the remaining discussions were conducted in Swahili.

### Job satisfaction

In general, health workers did not seem to be satisfied with their jobs. Even though almost all participants recognized that they appreciated and liked their job, they expressed dissatisfaction because of the low salary and difficult working conditions.

"It's not necessary that I'm not feeling happy; I can be feeling happy despite being paid little. But I'm not satisfied" (Nurse, Tumbi Hospital).

Clinical officers and medical attendants tended to be completely dissatisfied, which could be due to the fact that these two categories, in addition to low salary and heavy workload, suffer from an absence of job specification. They complained that they did not know what their duties were and were asked to do anything.

"This job is very hard and salary is low, and makes me very unhappy" (Medical Attendant, Kibaha Health centre).

### Salary

Almost 30% of all text was coded as "salary", showing how important this issue is for health workers. Even though a strike of health workers in 2005 resulted in pay's being improved by more than 70% for doctors, and by around 30% for nurses, clinical officers and medical attendants, salaries are still very low when compared to the cost of living and the inflation rate, especially for lower-ranked cadres. Indeed, salary was frequently reported as insufficient when compared to needs and workload.

"We are seriously living in a hard condition: salary itself can never sustain even food for the whole month, not to talk about school fees for our schoolchildren" (Health Worker, Dispensary of Mwendapole).

"On my side the work is good but the payment is very low comparing to the work we do" (Midwife, Tumbi Hospital).

Often health workers experienced consistent delays in payment or missing refunds of travel expenses. Moreover, salary was not perceived as adjusted for risks and for responsibilities.

### Coping strategies

When asked to discuss the strategies adopted in order to cope with the difficult situation, health workers mentioned the possibility of borrowing money, reducing expenses, relying on labour unions, running small private businesses, growing vegetables to sell at the market, doing extra jobs not in the health sector, working in the private sector as health professionals, and charging informally for services.

Moving to the private sector and especially to jobs with nongovernmental organizations was described as an opportunity, but only for doctors and nurses. This could help to explain the lower job satisfaction noticed among other cadres. However, participants reported that the private sector, even if it offers higher salaries and better working conditions, given the availability of drugs and equipment, lacks job security and is profit-oriented.

"For those who are running to the private sector, it is because of salary but some hate to be in the private sector simply because there is no job security, people are offended, there's no job satisfaction, there are tyrannies, and you can even lose the job anytime. Bad enough, in private sector you can learn some behaviours that we never expect from the public sector simply because they're profit minded" (Medical Doctor, Tumbi Hospital).

The issue of job security was found to be particularly important for health workers; some even stated that they would not have accepted even double salaries offered in the private sector because job security was not guaranteed. All groups raised and discussed this issue, even if it was not included in the focus group guidelines. The value given to job security can be due to the high unemployment rate and to the low recruitment rate in the public sector. In case of job loss, the possibility of being unemployed for a long time was seen as extremely high.

### Informal payments

Evidence of the existence and use of informal payments in the selected health facilities was confirmed, even if not every participant agreed that they themselves accepted informal payments. Sometimes participants recognized that the phenomenon may exist in other health facilities, but not in theirs (Tumbi Hospital). Different typologies of informal payments were described. Interestingly, participants frequently commented that unofficial payments were more commonly patient-initiated than provider-led:

"There are two different languages about this issue, one is when the health workers force the patients to give payments to them before service and this situation is not so common to happen, since the health workers will not be sure of the identity of the patients who could be a policemen or related. Second is when patients themselves force the health workers to receive gifts and money from them and this is very common. Most of the patients have the idea that to get a quality service then they should give the health workers something so that they will be much considered" (Clinical Officer, Tumbi Hospital).

The interpretation of this is quite tricky, as staff may wish to defend their integrity in the focus groups while defending the practice of charging. The fact that it is the patient offering the payment to the health worker may not exclude the compulsory nature of the fee. An "unwritten" rule known by both actors could be in operation. Nevertheless, the patient seems to be empowered by paying fees. Patients pay in order to obtain better or faster services, for example, by jumping the queue.

Staff and particularly doctors were also recommending patients to services or treatments in their own private practice:

"They [doctors] just tell them: this is the address of my clinic, come this afternoon, come tomorrow. This is the normal thing" (Nurse, Tumbi Hospital).

The amount taken as an informal payment seems to vary with the category of health worker. It is generally between 500 and 2000 Tanzania shillings (TZS; TZS 1000 = EUR 0.53) for medical assistants or nurses, but could be much higher for doctors and specialists, where amounts from TZS 10 000 to TZS 200 000 were mentioned.

Participants in all the groups recognized that the main reason for informal payments from the provider perspective was the inadequacy of salaries when compared to the needs of staff. All groups reported having difficulties in facing all the expenses until the end of the month, and even if they admitted not to be happy with the situation, they generally perceived the practice of asking for bribes as necessary and justified by the situation.

"Just imagine if a nurse or a doctor leaves nothing at home for that day. It's easy thing and normal for a person to ask for bribes in any places, so it is in the hospital. If one could be paid well and could provide for his family, he could live in peace and attend patients well" (Midwife, Tumbi Hospital).

Inadequate working conditions, disproportionate workload, absence of risk allowances or transport allowances and shortages of health care workers have also been mentioned as reasons to explain the practice from the providers' perspective.

The health care workers reported that sometimes informal payments were proposed by patients motivated by the belief of getting higher quality services or by the need or desire to be treated sooner.

Widespread corruption in the government, in the whole public sector and of top managers and clinic managers in the health sector was also mentioned as a factor that could justify and encourage the same practice to be adopted throughout the health system.

When asked to describe how the phenomenon could affect the health system, all participants agreed that access to health care was seriously compromised by this practice. It appears that poor people are unable to afford even primary care, while the majority of the population cannot access specialized services at all in urban public hospitals.

"Those who can't manage to bribe; they will all die since they will never be able to access the treatments. Just imagine the women who are about to deliver, or children and those who are really sick, or the poor: do you think they will survive? They will all die just for [inability to make] informal payments" (Midwife, Tumbi Hospital).

Very frequently health workers reported that not only access to but also quality of the health system was seriously compromised by this phenomenon. Informal payments create competition among health workers and induce dangerous, income-seeking behaviours and direct staff attention to profitable patients/services instead of necessary ones. Participants also mentioned that informal payments could damage the reputation of the category of health workers and of the hospitals involved.

### Informal payments, motivation and retention

The analysis of the perceived relationship between informal payments, and motivation and retention in the public health sector has yielded controversial and discordant results. For the majority of groups, informal payments were one of the strategies by which health workers cope with salaries that are sometimes below a reasonable level, but were not the reason for staying in the public sector.

"I just want to say that those who are engaged in informal payments are not happy of that. They are forced by a hard environment and their little income" (Health Worker, Dispensary of Mwendapole).

Many participants also described the circumstance in which, having paid a little bribe, the patient felt entitled to control the health worker through the entire episode of illness. This was the main demotivating factor for health workers in accepting informal payments.

"Patients themselves, after giving something, do control the health workers. You find that they don't respect the hospital regulations simply because of the favour that they gave to the health workers" (Doctor, Dispensary of Mwendapole).

"Bribery is a torture to health attendants, because even when you succeed to receive bribery from a person or patient then you will be locked to him, for he won't allow you to serve anyone else than him" (Midwife, Tumbi Hospital).

"To me, informal payments demotivate a health worker and destroy her/his performance because when the sick person will recognize that you ask them for informal payments they will make you a slave and control you. And even sometimes they might report the issue to the police and you'd be punished, including losing your job" (Health worker, Dispensary of Mwendapole).

The consequent "slavery" to the patient was perceived as humiliating and degrading.

"We are humiliated a lot if we will go on receiving bribes from patients, and also we will lose patients, which makes us not to be trusted, because there are many who are unable to give bribery. Those who are rich will get well, but those who are not capable will die earlier" (Midwife, Tumbi Hospital).

The fear of being caught or accused was another main source of discomfort and demotivation.

"When you receive bribery you become uncomfortable, you become afraid, you become insecure in such a way that sometimes when you hear a knock in your office you are afraid or if one talks about bribery you are confused. We don't like this situation, really it's not good" (Midwife, Tumbi Hospital).

The discomfort of being involved in a situation they knew had serious consequences for poor peoples' access to health services was also reported very frequently.

"On my side bribery is a trouble and a disturbance, and pity to those who are unable to give anything to be treated" (Midwife, Tumbi Hospital).

Another issue described was the competition that informal payments created among and within cadres. Since it is unlikely that a patient or his relatives can afford to bribe all the health workers involved in their treatment during an illness episode, they would have to select whom to corrupt; this was a source of rivalry and discontent.

However, in general other factors were more important in the decision to stay in the public sector. Job security was identified as the most important factor. In addition, opportunities for further education and training were considered important by certain categories, such as nurses and midwives. Clinical officers, instead, reported that the peculiarity of their category would not allow them to switch to the private sector or to migrate so easily. All categories, apart from specialists, conveyed that migration to other countries would have been difficult because they were not fluent in English, as Swahili was more commonly spoken and written.

As expected, given the reasons reported for the existence of informal payments, health workers' proposals on how to reduce the problem were all focused on incentives. The need to obtain salary increases was reported by all groups. After that, they mentioned the need to increase working conditions through better equipment, training opportunities and better management. The possibility of being compensated for risks and transport fees, night work and uniforms was often described as important in increasing job satisfaction and loyalty to the government, therefore decreasing illicit behaviour.

In addition, they mentioned the importance of implementing policies discouraging corruption in the entire public system by increasing accountability and transparency. Increasing patients' awareness of the issue and educating people to respect rules was also seen as crucial.

## Discussion

Informal payments have been reported to be very common in Tanzania in all levels of care and among all health worker categories. The amount paid varied depending on the services required and seemed to rise with the increase of specialization. These payments have been described as a coping strategy employed to respond to the circumstance of earning a salary that is insufficient to cover basic needs and incompatible with workload, responsibilities and risks taken. Based on the results of this study, it seems that informal payments are a form of financial incentive that also has substantial negative effects on motivation, contradicting the hypothesis of their possible positive impact on motivation.

**Figure 1:**
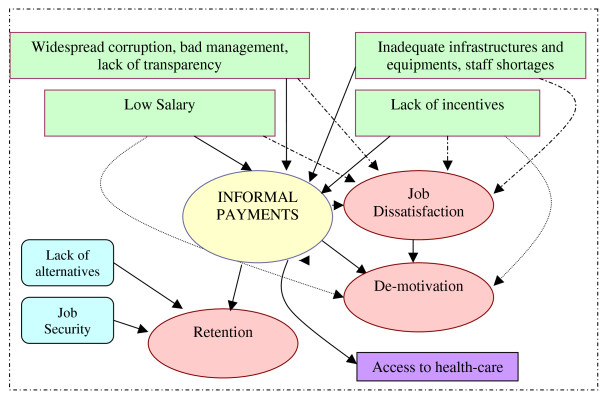
**A new framework for informal payments**.

A synthesis of the framework of informal payments as it emerged from this study is presented in Figure 1. A generally widespread discontent of health workers was observed. They appeared to be demotivated, dissatisfied and unhappy with their profession and their working environment. The same reasons behind job dissatisfaction were found to be behind the providers' justification of informal payments.

Surprisingly, though, based on the results of this study, it appears that most health workers are demotivated by accepting informal payments, rather than motivated. It seems that informal payments are asked for and accepted, but only because it is the easiest way of coping with a salary that is not in line with their needs and workload.

Informal fees, while increasing financial incentives, may at the same time lower motivation. In fact, informal payments are a particular form of financial incentive that is associated with other factors, such as fear of being detected, loss of self-esteem, a sense of guilt and humiliation.

Informal payments contribute to an environment of corruption and dishonesty that in turn creates dissatisfaction, discomfort and demotivation among health workers. This may be true for all cadres apart from doctors. This study suggest that doctors, and particularly specialists, practise dual jobs in the majority of cases; they often use their public job to maintain their reputation and recruit their patients for their private clinics. However, the practice of shifting patients from the public sector to the private sector is slightly different from charging informal fees for the provision of services offered in the public facilities, even if it can have the same effects for patients.

Results from this study suggest that informal payments contribute a little to retention in public health facilities, or they would not occur, but they do not contribute to motivation. Informal payments may help push health workers' salaries over the subsistence level, but at the expense of making them unhappier.

Nevertheless, there are other contributing factors to retention, including a context of high unemployment, temporary jobs and an absence of labour rights and some degree of public sector job security. In Tanzania, as noted, the lack of English fluency represents a great obstacle to migration.

Furthermore, certain health categories, such as clinical officers, medical assistants and nurse's aides, have been created with the special purpose of not being "exportable". Therefore, if other, more attractive jobs are not available, the benefits of staying in the public sector, however low, may seem higher than the alternatives, such as unemployment or subsistence farming.

This study had several limitations. The study was informative and exploratory, and the findings cannot be generalized outside of similar contexts. Moreover, possible biases could have occurred in the selection of participants. Participants were chosen randomly whenever possible, but the gatekeepers carried out the selection. It is therefore possible that the gatekeepers purposely chose more loyal health workers to preserve the reputation of the clinic. This may have led to an underestimation of the phenomenon of informal payments.

Furthermore, the need to divide by cadres and ensure the continuance of the activity in the clinics limited the number of health workers involved in the study, and this could have hampered the richness of the discussions. The generalizability of this study is limited by the size and methodology adopted. Results are therefore applicable only in similar contexts.

## Conclusion

This study suggests that, at least in this context, informal payments may demotivate health workers more than motivate them. This can be explained by negative factors involved with this practice, such as fear of detection, loss of self-esteem and sense of guilt, which counterbalance the positive financial incentives.

This study suggests that, with the possible exception of doctors, informal payments probably do not contribute much to the retention of health workers. On the contrary, other factors such as job security and lack of alternative employment may play a more important role in retaining staff.

As suggested by health workers in this study, the practice of informal payments contributes to the general demotivation of health workers and negatively affects access to health care services and quality of the health system. Even though further research is needed on the topic, the picture that has emerged requires urgent policy action. It is not just better financial incentives that are needed, but an environment in which corruption is endemic needs to be addressed, as it is believed that the reduction of informal payments will have a positive effect on access to and quality of health care.

## Competing interests

The authors declare that they have no competing interests.

## Authors' contributions

SS contributed to the conception and design of the study and acquisition and interpretation of data; performed main analyses; and wrote the original and successive drafts of the paper.

ST contributed to the conception and design of the study, contributed to interpretation of data and revised critically each draft. PB contributed to the conception and design of the study, contributed to interpretation of data and revised critically each draft. TM contributed to the design of the study and the acquisition of data. AM contributed to the conception and design of the study. All authors read and approved the final manuscript.

## Supplementary Material

Additional file 1 **Code list**. Table exceeding one A4 page in length. Click here for file
