# Supplementary material for: Understanding informal payments in health care: motivation of health workers in Tanzania
Source: Hum Resour Health. 2009 Jun 30;7:53. doi: 10.1186/1478-4491-7-53 (PMC2711965; doi:10.1186/1478-4491-7-53)
Supplement: Additional file 1 — Code list. Table exceeding one A4 page in length. [file 1478-4491-7-53-S1.doc]

| **CODE NAME** |
| --- |
| **Cadre** |
| *Salary and Motivation* |
| **Coping Strategies** |
| *Borrow* |
| *Complains* |
| *Extra jobs* |
| *Labour Unions* |
| *Reduce expenses* |
| *Work private sector* |
| **Incentives** |
| *Demotivation* |
| *Education and training* |
| *Motivation* |
| **Informal Payments** |
| *Answers* |
| Awareness |
| Good management |
| Incentives |
| Regulation |
| Salary raise |
| Working conditions |
| *Consequences* |
| *Access* |
| Danger - Illegal |
| Demotivation - Dissatisfaction |
| Discomfort |
| Motivation |
| Pressure from patient |
| Reputation of the category |
| Retention |
| *Reasons* |
| Ethics |
| Individual Behaviour |
| Need |
| Quality |
| Salary |
| Working conditions |
| Workload |
| *Typologies* |
| By the patient |
| Cadre |
| Poor |
| Rural and urban |
| *Where and Who* |
| By the patient |
| Cadre |
| Poor |
| Rural and urban |
| **Job Related Issues** |
| *Allowances* |
| *Job specification* |
| *Risk* |
| *Transfers* |
| *Transport* |
| *Uniforms* |
| **Job Satisfaction** |
| *Devotion* |
| *Mercy* |
| *Salary* |
| **Motivation** |
| *Demotivation* |
| *Motivation* |
| *Salary - motivation* |
| **Private Sector** |
| *Public – private* |
| *Shift to private - workers* |
| **Salary** |
| *Delays in payment* |
| *Experience* |
| *Increase* |
| *Migration* |
| *Motivation* |
| *Need* |
| *Working environment* |
| *Workload* |
| **System related Issues** |
| *Bad management* |
| *Corruption* |
| *Shortages* |
| **Working Environment** |
| *Migration* |
